# Improving peptide-MHC class I binding prediction for unbalanced datasets

**DOI:** 10.1186/1471-2105-9-385

**Published:** 2008-09-19

**Authors:** Ana Paula Sales, Georgia D Tomaras, Thomas B Kepler

**Affiliations:** 1 Center for Computational Immunology, Duke University, Durham, NC, 27705, USA; 2 Computational Biology and Bioinformatics PhD Program, Institute for Genome Sciences & Policy, Duke University, Durham, NC, 27705, USA; 3 Department of Biostatistics and Bioinformatics and Department of Immunology, Duke University, Durham, NC, 27705,USA; 4 Duke Human Vaccine Institute and Departments of Molecular Genetics and Microbiology, Immunology, and Surgery, Duke University, Durham, NC, 27705, USA

## Abstract

**Background:**

Establishment of peptide binding to Major Histocompatibility Complex class I (MHCI) is a crucial step in the development of subunit vaccines and prediction of such binding could greatly reduce costs and accelerate the experimental process of identifying immunogenic peptides. Many methods have been applied to the prediction of peptide-MHCI binding, with some achieving outstanding performance. Because of the experimental methods used to measure binding or affinity between peptides and MHCI molecules, however, available datasets are enriched for nonbinders, and thus highly unbalanced. Although there is no consensus on the ideal class distribution for training sets, extremely unbalanced datasets can be detrimental to the performance of prediction algorithms.

**Results:**

We have developed a decision-theoretic framework to construct cost-sensitive trees to predict peptide-MHCI binding and have used them to 1) Assess the impact of the training data's class distribution on classifier accuracy, and 2) Compare resampling and cost-sensitive methods as approaches to compensate for training data imbalance. Our results confirm that highly unbalanced training sets can reduce the accuracy of classifier predictions and show that, in the peptide-MHCI binding context, resampling methods do not improve the classifier performance. In contrast, cost-sensitive methods significantly improve accuracy of decision trees. Finally, we propose the use of a training scheme that, when the training set is enriched for nonbinders, consistently improves the overall classifier accuracy compared to cost-insensitive classifiers and, in particular, increases the sensitivity of the classifiers. This method minimizes the expected classification cost for large datasets.

**Conclusion:**

Our method consistently improves the performance of decision trees in predicting peptide-MHC class I binding by using cost-balancing techniques to compensate for the imbalance in the training dataset.

## Background

Determination of binding between peptide and Major Histocompatibility Complex class I (MHCI) is a crucial step in the development of subunit vaccines. The peptide-MHCI complexes are required for T cell activation and thus for the initiation of the adaptative immune response. Although MHCI binding does not alone determine the immunogenicity of peptides, it plays an important part, being a major bottleneck that separates immunogenic peptides from non-immunogenic ones. Hence, the ability to predict the binding between peptides and MHCI molecules would greatly reduce costs and accelerate the experimental process of identifying immunogenic peptides, which can then be used in the development of vaccines and therapies against neoplastic, infectious, and autoimmune diseases.

Our primary goal is to guide experimental research in identifying potential vaccine epitopes. In a given microbial genome, there are tens of thousands of peptides and the experimental assessment of the affinity between each peptide and an MHCI molecule represents a significant cost in terms of time and resources. The investigator has to consider the benefits of identifying binders versus the cost associated with experimentally testing nonbinders in order to decide which and how many peptides will be tested in the laboratory. This type of concern can be best addressed by the use of decision-theoretic approaches. Here we formalize such an approach to training decision trees to differentiate binders from nonbinders and show how costs that reflect this experimental tradeoff can be incorporated into the training of classifiers to increase their utility.

Myriad approaches have been applied to the prediction of peptide-MHCI binding. These methods can be divided into two broad categories: 1) MHCI structure-based methods, which use crystallized structures of MHCI molecules to develop computational models of the interaction between MHCI and peptides [1]; and 2) peptide sequence-based methods, which infer the physico-chemical preferences of a particular MHCI allele by analyzing the amino acid sequence of peptides with known affinity to it, where peptides with IC50 lower than a certain threshold, typically 500 nM [2], are classified as binders, and otherwise as nonbinders. Earlier prediction methods used the amino acid frequencies in each position of MHCI-eluted peptides to derive binding motifs and position specific scoring matrices (PSSMs). Methods of this type include SYFPEITHI [3] and BIMAS [4], which have been publicly available and used extensively by the experimental community. As the number of peptides in the MHCI databases increased, so did the number of different machine learning methods that were applied to this problem, which include artificial neural networks [5], support vector machines [6], hidden Markov models [7], Gibbs sampling [8], and classification trees [9-11].

While some of these methods achieve outstanding performance in predicting binding between peptides and certain MHCI alleles, all of them suffer from the fact that the available data for training is heavily biased towards one class of peptides (either binders or nonbinders). There is a vast literature on the impact of class distribution of training sets on the performance of the prediction algorithms (for further readings see Chawla *et al*., 2004 [12]), and although there is not a straightforward answer to the question of what the ideal class distribution of training datasets is, it has been suggested that a balanced distribution or the estimated distribution of the target population should be used. Moreover, it is a well known phenomenon that highly unbalanced datasets are detrimental to classifier performance. The imbalance in the peptide-MHCI binding data depends on the experimental methods used to produce them: either elution assays, in which case the dataset consists purely of binders; or binding assays in which peptides are tested for binding or affinity to a particular MHCI allele, leading to datasets consisting mostly of nonbinders. The reason for this imbalance towards nonbinders is that binders are extremely rare in nature: It has been estimated that the proportion of peptides in a protein that will bind to a given MHC allele varies between 0.001 and 0.05 [13]. Datasets generated in different laboratories using different assays and conditions are often inconsistent with each other and thus combination of datasets can be very difficult.

Here we investigate how best to use unbalanced datasets to train algorithms for the prediction of peptide-MHCI binding. Although there is no universally agreed upon method for dealing with unbalanced data, several techniques have been proposed to deal with this issue and have been demonstrated to improve prediction accuracy depending on the context in which they are used [12]. Elkan [14] showed how to make a standard learning algorithm yield cost-sensitive results when trained with an unbalanced dataset. Another successful strategy is referred to as cost-sensitive methods, in which weights are used to compensate for the imbalance in the ratio of the two classes. Other methods pre-process the data to achieve a balanced class distribution. In particular two resampling methods stand out: 1) Undersampling, where random cases of the majority class are deleted until both classes have the same number of cases; and 2) Oversampling, where random cases of the minority class are duplicated until both classes have the same number of cases. Our primary goal is to determine whether or not the accuracy of peptide-MHCI binding prediction can be improved by the use of methods that compensate for the training data imbalance, such as resampling and cost-sensitive methods. The results presented herein suggest that resampling procedures, such as undersampling and oversampling, do not consistently improve the utility of classifiers used in the context of peptide-MHCI binding. The cost-sensitive method, however, significantly improves prediction accuracy when the training data is biased towards nonbinders. These results are derived from analysis using decision trees. The underlying mathematical treatment is, however, quite general, and can be applied to any classifier capable of cost-sensitive learning, including most of the classifiers used in peptide-MHCI binding prediction.

## Methods

### Approach

The development of subunit vaccines is a multi-step process; at each stage, the investigators must decide whether a particular peptide warrants further investment or should be omitted from further experimentation. These decisions must be informed, either explicitly or implicitly, by consideration of the costs incurred in continuing the experiments and of the potential reward for a positive discovery. One must also estimate the probability that a given decision will be erroneous, either as a false positive (continuing to invest in a peptide that will prove to be unsuitable) or a false negative (discontinuing tests on a peptide that would have worked). Let the cost of misclassifying a binder be denoted *κ*_2 _(for type 2 error) and that for misclassifying a non-binder, *κ*_1_. We refer to *κ*_2 _as the "real-world" cost, as it can be interpreted as the number of nonbinders an investigator is willing to test in the laboratory in order to find one binder. Finally, suppose that we can parameterize a family of classifiers with the continuous vector *θ*. Then the cost, *K*, incurred in making a decision on a peptide *ϕ *using the classifier *T*(*θ*) is

(1) *K*(*ϕ*|*θ*) = *τ*_+_(*ϕ*)*κ*_2_*c*_-_(*ϕ*|*θ*) + *τ*_-_(*ϕ*)*κ*_1_*c*_+_(*ϕ*|*θ*),

where *τ*_+ _and *τ*_- _are indicators of true class and *c*_+_(·|*θ*) and *c*_-_(·|*θ*) are indicators of the classification induced by *T*(*θ*). The expected decision cost over all peptides is

(2) $$EK\left(\theta \right)=\pi k_{2}\in_{2}\left(\theta \right)+\left(1-\pi \right)k_{1}\in_{1}\left(\theta \right)$$

where *π *is the proportion of binders in the population and *ϵ*_*i *_is the expected rate of type *i *errors. We would like to find the classifier T* that minimizes this expected cost. In the training context, we use the "training cost function", *K*_*T*_(*ϕ*|*θ*), which has the same form as the decision cost described above, but differs from it in the fact that both the false positive *λ*_2 _and false negative *λ*_1 _costs are now tunable parameters:

(3) *K*_*T*_(*ϕ*|*θ*) = *τ*_+_(*ϕ*)*c*_-_(*ϕ*)*λ*_2 _+ *τ*_-_(*ϕ*)*c*_+_(*ϕ*)*λ*_1_.

Because we only ever have access to finite datasets to train classifiers, the training cost from using such classifiers can be decomposed into two parts: the decision-making cost described in Eq. 1 and the residual, due to the deviation of the training set *D *from the whole population:

(4) $$\begin{array}{lll} K_{T}(D|\theta ) & = & n\{p\lambda_{2}\epsilon_{2}(\theta )+(1-p)\lambda_{1}\epsilon_{1}(\theta )\}\\ & + & \sum_{\phi \in D}\left\{\tau_{+}(\phi )\lambda_{2}\delta c_{-}(\phi |\theta )+\tau_{-}(\phi )\delta c_{+}(\phi |\theta )\right\} \end{array}$$

where *n *is the size of the training dataset, *p *is its proportion of positives and the classification error is defined on positive (negative) peptides as

(5) *δc*_-(+)_(*ϕ*|*θ*) ≡ *c*_-(+)_(*ϕ*|*θ*) - *ϵ*_2(1)_(*θ*)

We may further abbreviate this expression to

(6) *K*_*T*_(*D*|*θ*) = *n *{*pλ*_2_*ϵ*_2_(*θ*) + (1 - *p*)*λ*_1_*ϵ*_1_(*θ*)} + *R*(*D*; *θ*, *λ*).

The expected decision cost described in Eq. 2 is minimized at $\hat{\theta }$ where

(7) $$0=\frac{\partial }{\partial \theta }EK(\hat{\theta })=\pi \kappa_{2}\frac{\partial \epsilon_{2}}{\partial \theta }(\hat{\theta })+(1-\pi )\kappa_{1}\frac{\partial \epsilon_{1}}{\partial \theta }(\hat{\theta }).$$

Similarly, the training cost function is minimized at θ * where

(8) $$0=\frac{\partial }{\partial \theta }L(D|\theta^{*})=p\lambda_{2}\frac{\partial \epsilon_{2}}{\partial \theta }(\theta^{*})+(1-p)\lambda_{1}\frac{\partial \epsilon_{1}}{\partial \theta }(\theta^{*})+\frac{\partial R}{\partial \theta }(D;\theta^{*},\lambda ).$$

Denote the value of *θ *that minimizes the expected decision cost by $\hat{\theta }$, and that that minimizes the training cost function by θ * = $\hat{\theta }$ + 1/*n δθ*. Now differentiation and Taylor expansion yield the sufficient condition for the minimum of the training cost function to approach $\hat{\theta }$ as *R*(*θ*)/*n *→ 0:

(9) $$\lambda_{2}^{B}=\frac{\pi }{1-\pi }\frac{1-p}{p}\frac{\kappa_{2}}{\kappa_{1}}\lambda_{1}.$$

**Figure 1:**
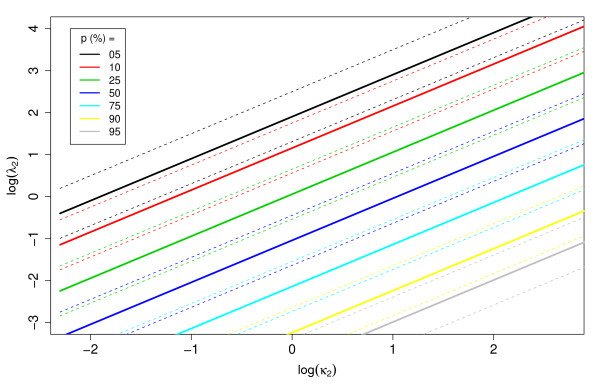
**Theoretical relation between *λ*_2 _and *EK*(*θ*)**. Theoretical relationship between the training false negative cost (*λ*_2_) that minimizes the expected cost of a classifier (*EK*(*θ*)) for a given type 2 error cost (*κ*_2_). The dotted lines represent one standard deviation from the mean. Here *κ*_1 _= 1, *λ*_1 _= 1 and *π *= 0.5.

This expression defines what we refer to as the "balancing cost", $\lambda_{2}^{B}$. The basic intuition behind the balancing cost is that its use results in both classes having equal importance in the training of the classifier. It is helpful to note that: 1) As the real-world false negative cost *κ*_2 _increases, so does the balancing cost; and 2) As the proportion of positives *p *in the training set increases in relation to the population positive frequency *π*, the balancing cost decreases (see figure 1). Finally, we have

(10) $$\delta \theta =-\frac{\partial R}{\partial \theta }{\left[p\lambda_{2}\frac{\partial^{2}\epsilon_{2}}{\partial \theta^{2}}+(1-p)\lambda_{1}\frac{\partial^{2}\epsilon_{1}}{\partial \theta^{2}}\right]}^{-1},$$

with the right-hand side evaluated at $\hat{\theta }$, which provides a first-order correction for finite datasets. Figure 1 displays the relation between population and training sample positive proportions and costs as described in Eq. 9 and can serve as a guideline of what weights to assign to peptides of different classes given the class distribution in the training set and the relative importance of positives versus negatives in the real-world application.

### Datasets

The peptide binding data used to train and test the decision trees were obtained from a publicly available database published by Peters *et al*. [2], where the peptide affinity to a particular MHCI molecule is measured by one of two assays, and classified as binder when its IC50 is less or equal to 500 nM, and nonbinder otherwise. Decision trees were constructed for each one of 35 alleles in the dataset. The cost-sensitive and resampling experiments (described below) were performed for five alleles: A0203, A1101, A3101, B0702 and B1501. The numbers of peptides in the datasets for these five alleles are shown in table 1.

**Table 1:** Number of binders and nonbinders in Peters *et al*. [2] datasets for 5 alleles.

allele	binders	nonbinders
A0203	639	804
A1101	695	1290
A3101	427	1442
B0702	210	1052
B1501	179	799

### Cost Adjustments

Seven training sets for each allele studied were generated, such that all training sets for a given allele had the same number of observations but varying proportions *p *of positives, namely 5%, 10%, 25%, 50%, 75%, 90% and 95%. These training sets were created as follows. First, 25% of the binders and 25% of the nonbinders were randomly selected and set aside as a testing set. The remaining 75% of the binders and of the nonbinders formed the "training superset", from which the peptides for the various training sets were sampled. The total number of peptides in each of the seven training sets was fixed and equal to the number of peptides of the minority class in the training superset. The minority class was the positive for all 5 alleles that we tested. Finally, the training sets were formed by randomly sampling without replacement positive and negative peptides from the training superset such that the described class distribution was reached. The numbers of binders and nonbinders in the resulting training sets are shown in table 2.

**Table 2:** Training sets used in the cost-sensitive experiment

	A0203		A1101		A3101		B0702		B1501	
%pos	B	NB	B	NB	B	NB	B	NB	B	NB

05	24	456	26	495	16	304	7	150	6	128
10	48	432	52	469	32	288	15	142	13	121
25	120	360	130	391	80	240	39	118	33	101
50	240	240	261	261	160	160	79	79	67	67
75	360	120	391	130	240	80	118	39	101	33
90	432	47	469	52	288	32	142	15	121	13
95	456	24	495	26	304	16	150	7	128	6

Number of binders (B) and nonbinders (NB) in training sets.

The goal of this set of experiments was two-fold: 1) To investigate the relationship between class distribution and classifier performance, and 2) To learn how can misclassification costs be used to improve prediction accuracy for a given class distribution of the training set. We emphasize that our goal is not to improve upon existing computational methods, but rather to show that the performance of a single classifier can be improved with the use of cost-sensitive techniques. Misclassification costs were used as weights with the purpose of artificially changing the class distribution of the training dataset. The false negative cost (*λ*_2_) can be interpreted as the weight given to the peptides in the positive class, and similarly false positive cost (*λ*_1_) is the weight given to the negative class. The overall scale of the training cost function (Eq. 6) is arbitrary, so we have fixed *λ*_1 _= 1 and varied *λ*_2 _between 1/20 and 20 in order to investigate the relationship between costs and class distribution.

Previous works (e.g., [15]) have suggested that among the best class distributions for learning is the balanced distribution, one in which all classes are equally represented. We assume that given an unbalanced training set, a balancing misclassification cost can be used to achieve an artificially nearly-balanced class distribution. The balancing cost, $\lambda_{2}^{B}$, defined in Eq. 9 can be interpreted to be the *λ*_2 _that weights the positive peptides to be the same number as the negatives and therefore compensates for the imbalance ratio of the two classes. Consider the simplest scenario, where *λ*_1 _= 1, *κ*_1 _= 1, *κ*_2 _= 1 and *π *= 0.5, then the balancing cost reduces to

$$\lambda_{2}^{B}=\frac{\left(1-p\right)}{p}$$

We are particularly interested in how classifiers trained with this simplified balancing cost perform compared to the best classifiers for a given allele, as well as compared with classifiers trained with unit costs (*λ*_1 _= 1 and *λ*_2 _= 1).

### Resampling

#### Undersampling

The undersampling method consists of randomly eliminating peptides of the majority class from the training set until both classes have the same number of examples. The training sets were constructed in a similar manner to the cost-modifying experiment. First, we set aside 25% of binders and nonbinders into the testing set. The remaining binders were put into the training set together with the same number of nonbinders, which were randomly sampled without replacement from the nonbinders training superset. One of the issues concerning undersampling is the loss of information that results from the process, which can be aggravated when particularly important elements are removed from the training set. To get around this problem, we used 10-fold crossvalidation and the results presented here are the average of the 10 experiments.

#### Oversampling

The oversampling method consists of randomly replicating peptides of the minority class into the training set until both classes have the same number of examples. The training sets were constructed as follows. First, we set aside 25% of binders and nonbinders into the testing set. All remaining peptides were put into the training set together with *d *peptides from the minority class which were sampled with replacement, where *d *is the difference between the number of peptides in the the training set belonging to the majority and minority classes. Hence, each peptide of the minority class is represented at least once and possibly multiple times in the training set. Similarly to the undersampling procedure, we used 10-fold crossvalidation and the results presented here are the average of the 10 experiments.

### Decision trees

The present study applies tree-based models to the peptide-MHCI binding prediction problem. We have chosen to use decision trees for the simplicity in their interpretation and also because they have not been thoroughly explored in the context of peptide-MHCI binding. Moreover, decision and classification trees have become the canonical method for comparison of techniques used to deal with unbalanced datasets in the machine learning community. Finally, there seems to be a natural correspondence between the importance of the different residue positions in a peptide and the hierarchical way in which decision trees are constructed.

#### Tree generation

Breiman *et al*. [16] provides an excellent and detailed description of classification and regression trees. Briefly, given a dataset in which each object, *φ*, is represented by a (*τ *(*φ*), **x**(*φ*)) pair, where **x**(*φ*) is a vector containing attributes of the object and *τ *is an indicator function of the class of the object, a tree-based classifier recursively partitions the data's attribute space into sub-regions, called *nodes*, in which the response variable is increasingly more homogeneous. These trees are created in two steps: (1) induction of a large tree; and (2) pruning of the large tree into gradually smaller subtrees (here we use the cost-complexity pruning [16]). Finally, one subtree must be chosen from the sequence of subtrees generated by the pruning process. In the present study, we chose the tree that minimizes the training cost function (Eq. 6) when applied to the test set.

The construction of a tree requires (1) a set of *splits*, which are binary questions with mutually exclusive and exhaustive outcomes used to partition the data, where the questions are coined in terms of the attributes of the objects in the dataset; and (2) a *split function *used to quantify the goodness of a split, by measuring the change in the homogeneity of the response variable in the tree due to splitting a node into two subsets based on the given split.

#### Splits and split function

In the problem at hand, the training dataset consists of peptides *ϕ*, where *τ*(*ϕ*) is the class of the peptide (either binder or nonbinder) and **x**(*ϕ*) is the linear sequence of amino acids of the peptide, with *x*_*j *_being the *j*^*th *^amino acid from the amino terminal end of the peptide. The binary questions about the sequence of peptides can be phrased in several distinct ways, and each one of them generates a different class of splits, called *motifs*, that can be used in the construction of trees. We used motifs based on the anchor positions, which are represented by a single amino acid with a fixed position in the peptide, such that every amino acid is represented in every position of the peptide. The amino acids, in turn, can be represented in one of two ways: 1) by the traditional amino acid single-letter code. For example, alanine is represented by "A", arginine by "R" and so forth; and 2) by their physico-chemical properties, namely molecular weight, hydropathicity, volume, isoelectric point, polarity, ability to form hydrogen bonds and chain type (aliphatic, aromatic) as previously shown [17].

The split function used was the training cost described in Eq. 3.

## Results

### Cost Adjustments

**Figure 2:**
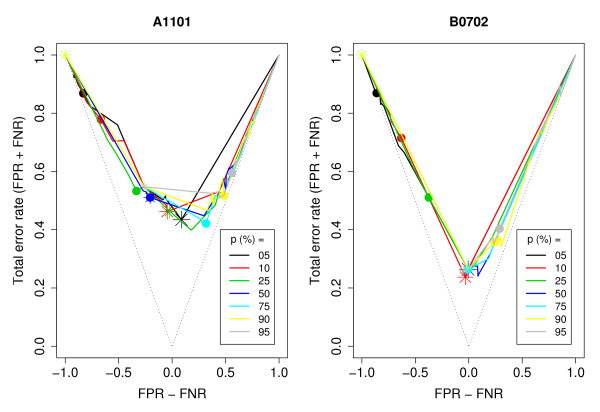
**Classifier performance vs. class distribution**. Comparison of the performance of classifiers built with training sets of same size but different proportions of positives for alleles A1101 (left panel) and B0702 (right panel). Each point in a curve represents a classifier constructed with a different false negative training cost. The classifier constructed with the unit cost (*λ*_2 _= 1) in each curve is marked with a solid circle and that constructed with the balancing cost is marked with a star. The curve for the perfect classifier would lie on the dotted line. The y-axis shows the total error rate of a classifier, which is the same as the classifier cost (*K*) when the type 1 and type 2 misclassification costs are identical (*κ*_1 _= *κ*_2 _= 1). FNR: false negative rate. FPR: false positive rate.

The first goal of this set of experiments was to investigate the relationship between class distribution and classifier performance. Our results suggests that for a fixed training set size, decision trees perform best when trained with datasets of nearly balanced class distribution. Figure 2 shows the performance of classifiers trained with datasets of the same size but different class distributions and training costs for alleles A1101 and B0702 (see Additional file 1 for the results for the other three alleles). Note that as the proportion of positives in the training set increases, the false negative rate decreases and the false positive rate increases as can be seen by the subtle shift in the curves from left to right.

Our second goal was to determine whether or not prediction accuracy of a given classifier can be improved by the use of cost-sensitive techniques and, if so, to establish the relationship between classifier performance and training costs. Our results demonstrate that misclassification costs can be used to improve prediction accuracy. In fact, for each one of the alleles we tested there was a cost *λ*_2 _that performed significantly better than the unit cost, as can be seen by the increase in AUC shown in table 3. Although our goal is not to improve upon the performance of existing methods, we also show in table 3 the AUC for 4 other methods as described in [2] for purposes of comparison.

**Table 3:** Comparison of classifiers performance as measured by AUC

allele	Trees, unit *λ*_2_	Trees, best *λ*_2_	ARB*	SMM*	ANN*	External Tool*
A0101	.903	.951	.964	.980	.982	.955
A0201	.796	.842	.934	.952	.957	.922
A0202	.770	.770	.875	.899	.900	.793
A0203	.746	.781	.884	.916	.921	.788
A0206	.732	.747	.872	.914	.927	.735
A0301	.763	.763	.908	.940	.937	.851
A1101	.841	.859	.918	.948	.951	.869
A2301	.742	.782	NA	NA	NA	NA
A2402	.685	.748	.718	.780	.825	.770
A2403	.673	.846	NA	NA	NA	NA
A2601	.606	.811	.907	.931	.956	.736
A2902	.783	.847	NA	NA	NA	NA
A3001	.741	.861	NA	NA	NA	NA
A3002	.777	.810	NA	NA	NA	NA
A3101	.825	.833	.909	.930	.928	.829
A3301	.636	.827	.892	.925	.915	.807
A6801	.756	.761	.840	.885	.883	.772
A6802	.699	.714	.865	.898	.899	.643
A6901	.614	.813	NA	NA	NA	NA
B0702	.887	.911	.952	.964	.965	.942
B0801	.547	.835	.936	.943	.955	.766
B1501	.759	.823	.900	.952	.941	.816
B1801	.745	.833	.573	.853	.838	.779
B2705	.753	.892	.915	.940	.938	.926
B3501	.712	.771	.851	.889	.875	.792
B4001	.587	.897	NA	NA	NA	NA
B4002	.718	.778	.541	.842	.754	.775
B4402	.588	.762	.533	.740	.778	.783
B4403	.647	.804	.461	.770	.763	.698
B4501	.679	.824	NA	NA	NA	NA
B5101	.664	.792	.822	.868	.866	.820
B5301	.795	.819	.871	.882	.899	.861
B5401	.654	.796	.847	.921	.903	.799
B5701	.756	.936	.428	.871	.826	.767
B5801	.815	.864	.899	.964	.961	.899

The second and third columns correspond to the decision trees described in the present work. Note the improvement in the performance of the trees with the use of training costs. *Values extracted from table 2 of Peters *et al*., 2006.

**Figure 3:**
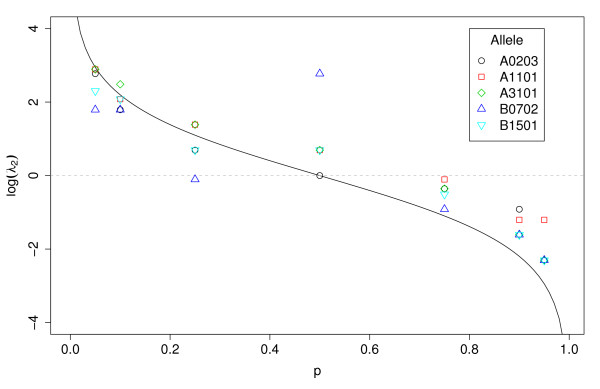
**Balancing cost vs. minimizing cost**. Comparison of balancing cost (solid black line) and the minimizing costs (symbols) for each one of the five alleles.

Note in figure 2 that for the training sets with majority of nonbinders, $\lambda_{2}^{B}$ consistently reduced the total error rate as compared to the unit cost (*λ*_2 _= 1). The impact of $\lambda_{2}^{B}$ on the performance of classifiers trained with binders-enriched datasets was not consistent, being better than unit cost for some classifiers and worse for others. In addition to representing an improvement over the unit cost, in a few cases $\lambda_{2}^{B}$ coincided with the minimizing cost, that is, the most accurate classifier for a given allele and training set was the one trained with $\lambda_{2}^{B}$. However, in most cases, the balancing cost over-compensated for the imbalance in the class distribution, such that it was larger than the minimizing cost (see figure 3)

**Figure 4:**
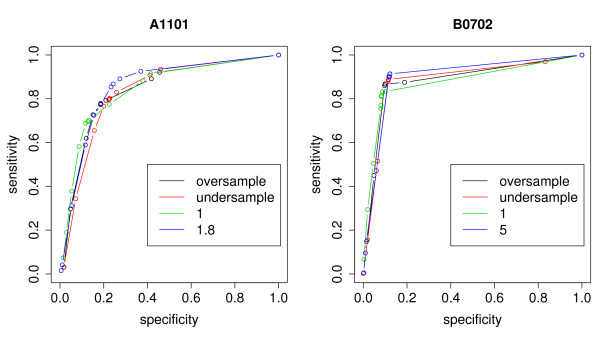
**Comparison of unit cost, balancing cost, undersampling and oversampling**. ROC curves for alleles A1101 (left panel) and B0702 (right panel) comparing the results of trees constructed with the oversampled training set (black curve), the undersampled training set (red curve), and the full training set without training costs, that is, *λ*_1 _= *λ*_2 _= 1 (green curve) and with the balancing training cost, that is, *λ*_1 _= 1 and *λ*_2 _= (1 - *p*)/*p *(blue curve). The ROC curves were constructed by varying the threshold used to label a node from 0 to 1 and evaluating its sensitivity and specificity at each threshold.

We then compared the performance of trees trained with the complete dataset using either the unit cost or $\lambda_{2}^{B}$ (the red and green ROC curves in figure 4, respectively). The use of $\lambda_{2}^{B}$ resulted in AUC at least as large as those for unit cost, such that $\lambda_{2}^{B}$ improved the ROC curves as compared to the unit cost in the majority of the cases. One interesting feature of the use of $\lambda_{2}^{B}$ is that it consistently shifts the ROC curve toward increasing sensitivity at the price of decreasing specificity, which is a desirable tradeoff when binders are rare. Thus, even in the cases when the increase in AUC is not substantial, the use of $\lambda_{2}^{B}$ can still represent an improvement over unit cost due to the shift it causes to the ROC curve.

### Resampling

The results obtained using the balanced undersampled and oversampled training sets did not represent an improvement over those using the complete unbalanced training sets (see figure 4 and Additional file 2). For alleles A0203, A1101 and B0702, the ROC curves for the trees trained with the entire dataset and those trained with the re-sampled dataset were indistinguishable from one another, whereas for alleles A3101 and B1501, the use of undersampling severely damaged the accuracy of the trees.

### Real-world costs versus training costs

**Figure 5:**
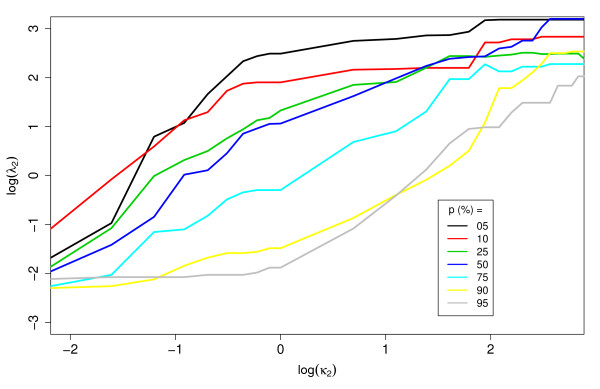
**Empirical relation between ${\hat{\lambda }}_{2}$ and *EK*(*θ*)**. Optimal false negative training cost (${\hat{\lambda }}_{2}$) as a function of type 2 error cost (*κ*_2_). Classifiers were trained at multiple values of ${\hat{\lambda }}_{2}$ and tested at *κ*_2 _(compare with figure 1). This was done for each of the five alleles and the *λ*_2 _shown in the curves are the average of the minimizing *λ*_2 _for each allele.

We built decision trees with the training data described in table 1 using different values of false negative cost (*λ*_2_), and evaluated them on a test set using the "real-world" cost *κ*_2_. We call ${\hat{\lambda }}_{2}$ the training cost that minimizes the total cost of a classifier on the test set for a given *κ*_2_. Figure 5 shows the relationship between ${\hat{\lambda }}_{2}$ and *κ*_2_. Note that although the results are relatively noisy, in general the same trend shown in theory can be observed from this empirical data (see figure 1). The ${\hat{\lambda }}_{2}$ increases with *κ*_2 _and as the proportion of positives in the training set increases, the line shifts to the right, indicating that for a particular value of *κ*_2_, the suggested ${\hat{\lambda }}_{2}$ decreases.

## Discussion

Prediction of peptide-MHCI binding has great potential to accelerate and reduce the cost of subunit vaccine development. One of the issues concerning the prediction of MHC-peptide binding is that binders are much less abundant than nonbinders, and thus much harder to find experimentally. This circumstance typically leads to highly unbalanced training sets, which can hinder the performance of algorithms trained with them. In fact, such training sets lead to a significant increase of type 2 errors and thus make it more difficult still to find binders.

Our results show that highly unbalanced training sets do indeed reduce the accuracy of predictions made with decision trees and that these predictions improve as the training sets become more balanced. We have examined three approaches that aim at improving classifier accuracy by compensating for the imbalance in the class distribution of the training sets: undersampling, oversampling and a cost-sensitive method. Overall, resampling did not improve the performance of the decision trees. In fact, in several cases classifiers trained with undersampled training sets performed much worse than those trained with the full dataset. This could have been caused by the loss of information relevant to the training process. For this reason, undersampling methods may only be appropriately used with datasets in which the majority class contains a lot of redundancy, in which circumstance undersampling has been shown to outperform other random resampling methods in four distinct datasets [18]. Another potential drawback of undersampling, and in broader terms of random resampling methods, is that they may yield noisy results due to the variability introduced in the process by the randomness of the sampling procedure.

In contrast to undersampling, using misclassification costs as a means to artificially counterbalance data bias led to significant improvements in the performance of the decision trees in the majority of the cases. Although cost-sensitive procedures do not add any extra information to the training set, they seem to be more advantageous than random resampling techniques because they do not cause loss of information as does undersampling and do not have the extra variability introduced by the random sampling process. Several other studies have shown cost-modifying methods to be advantageous. For example, Japkowicz and Stephen [19] performed a systematic comparison of these methods in both artificially-generated and real-world domains, showing that cost-modifying methods yield better results than resampling techniques. Fundamentally, the cost-sensitive method described here can be straightforwardly applied to any classifier that is trained using datasets that include both classes of peptides, binders and nonbinders. For instance, the individual weights of a weight matrix can be derived by minimizing the cost function (Eq. 3) over these weights. The indicator function *c*_-(+)_(*ϕ*) can be defined by the score function's being above or below a given threshold, where the scoring function is typically the sum of the scores of each amino acid in each position of a peptide. Similarly, this cost function can be incorporated into a neural network by differentially weighting the output depending on the class of the training example and allowing it to be used in the learning process by the backpropagation procedure [20,21]. Likewise, for support vector machines, the cost function can be implemented through the definition of the "soft margin" [22], allowing the SVM to misclassify more examples of one class than examples of the other class.

In addition to showing that peptide-MHCI binding predictions can be improved by the use of cost-sensitive decision trees, we have investigated the use of the balancing cost, $\lambda_{2}^{B}$, as a rule-of-thumb to train classifiers. We have shown that although $\lambda_{2}^{B}$ is not always the *λ*_2 _that minimizes the total cost of the classifier, it consistently outperforms the unit cost (*λ*_2 _= *λ*_1_) when the training set is enriched for nonbinders.

Moreover, we have showed that the use of $\lambda_{2}^{B}$ shifts the ROC curves towards areas of higher sensitivity in relation to ROC curves generated with unit cost, which can be highly desirable in situations such as epitope discovery projects.

Thus, although the relationship between training costs and class imbalance is relatively noisy, and further studies should be conducted before a complete guideline of what training costs should be used for a particular peptide-MHCI binding dataset, our results allow us to suggest that a balancing cost should be used for datasets enriched for nonbinders, and the unit cost should be used for binders-enriched training sets.

## Conclusion

The vaccine development process is costly and time-consuming, requiring decisions to be made at each step and it lends itself nicely to a decision-theoretic approach, which we have described here. In particular, at the epitope discovery stage, there are real costs associated with the risk of missing a positive and with the experimental verification of nonbinders. Here we have described a decision-theoretic framework for the prediction of peptide-MHCI binding and have provided a guideline on how to incorporate real-world costs together with misclassification costs at the training level in order to maximize prediction accuracy and push it in the desired direction.

## Authors' contributions

APS performed the computational experiments, analyzed the data and wrote the manuscript. TBK supervised the study and wrote the manuscript. GDT supervised the pilot experimental studies and helped revise the manuscript.

## Supplementary Material

Additional file 1 Classifier performance vs class distribution for alleles A0203, A3101 and B1501. Comparison of the performance of classifiers built with training sets of same size but different proportions of positives for alleles A0203, A3101 and B1501 (compare to figure 2). Each point in a curve represents a classifier constructed with a different false negative training cost. The classifier constructed with the unit cost (*λ*_1 _= 1) in each curve is marked with a solid circle and that constructed with the balancing cost is marked with a star. The curve for the perfect classifier would lie on the dotted line. The y-axis shows the total error rate of a classifier, which is the same as the classifier cost (*K*) when the type 1 and type 2 misclassification costs are identical (*κ*_1 _= *κ*_2 _= 1). FNR: false negative rate. FPR: false positive rate. Click here for file

Additional file 2 Comparison of unit cost, balancing cost, undersampling and oversampling for alleles A0203, A3101 and B1501. ROC curves for alleles A1101 (left panel) and B0702 (right panel) comparing the results of trees constructed with the oversampled training set (black curve), the undersampled training set (red curve), and the full training set without training costs, that is, *λ*_1 _= *λ*_2 _= 1 (green curve) and with the balancing training cost, that is, *λ*_1 _= 1 and *λ*_2 _= (1 - *p*)*/p *(blue curve). Compare to figure 4. The ROC curves were constructed by varying the threshold used to label a node from 0 to 1 and evaluating its sensitivity and specificity at each threshold. Click here for file
