# Supplementary figures and images for: Improving peptide-MHC class I binding prediction for unbalanced datasets
Source: BMC Bioinformatics. 2008 Sep 19;9:385. doi: 10.1186/1471-2105-9-385 (PMC2586639; doi:10.1186/1471-2105-9-385)

A0203

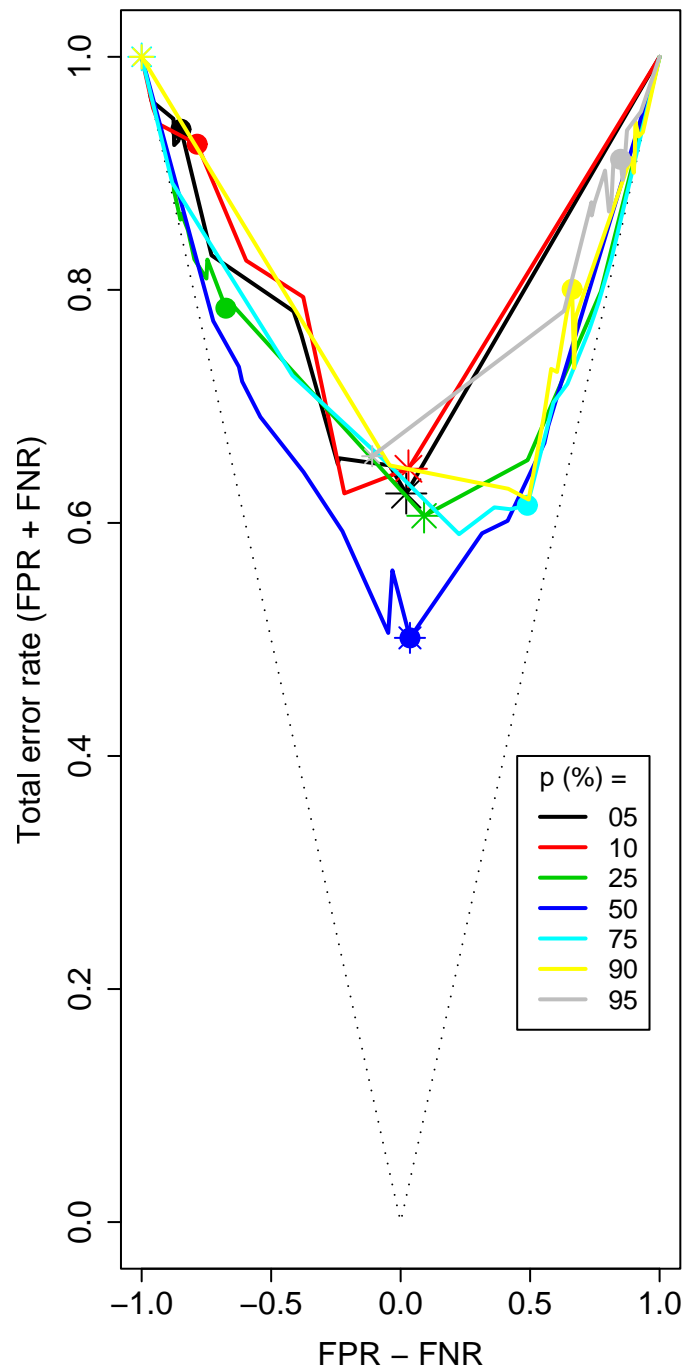

A3101

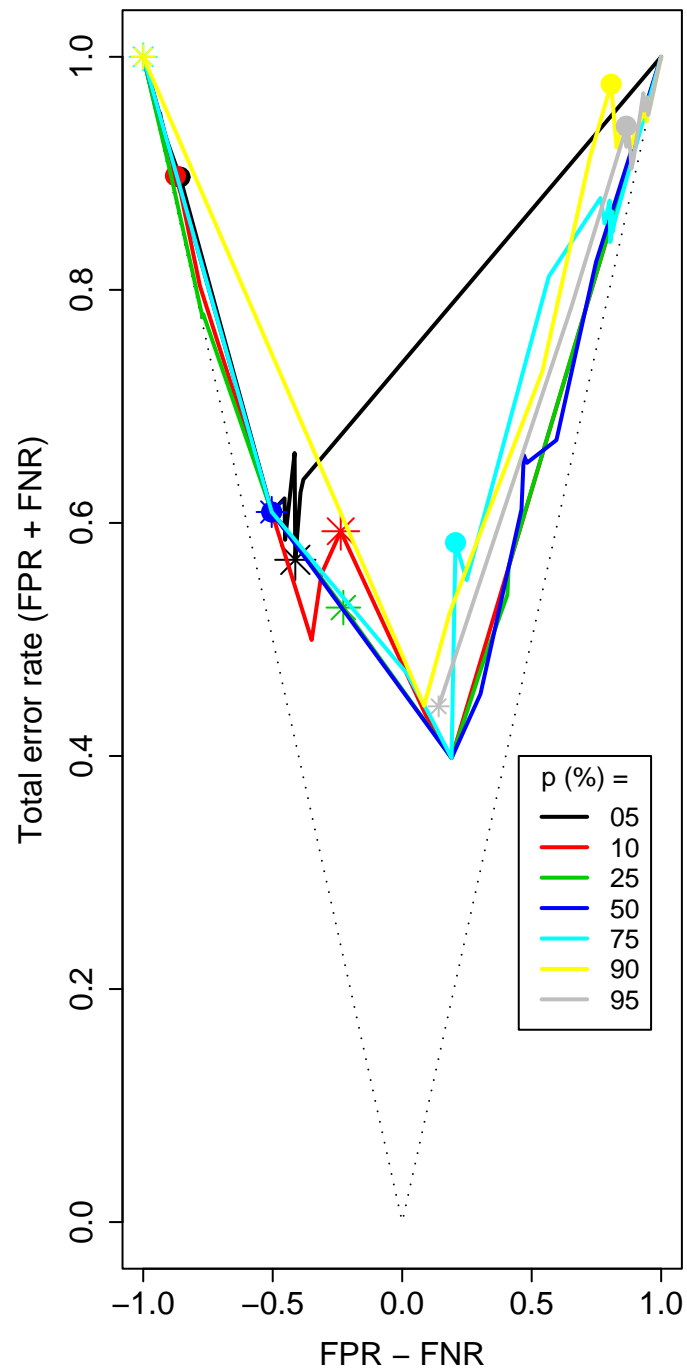

B1501

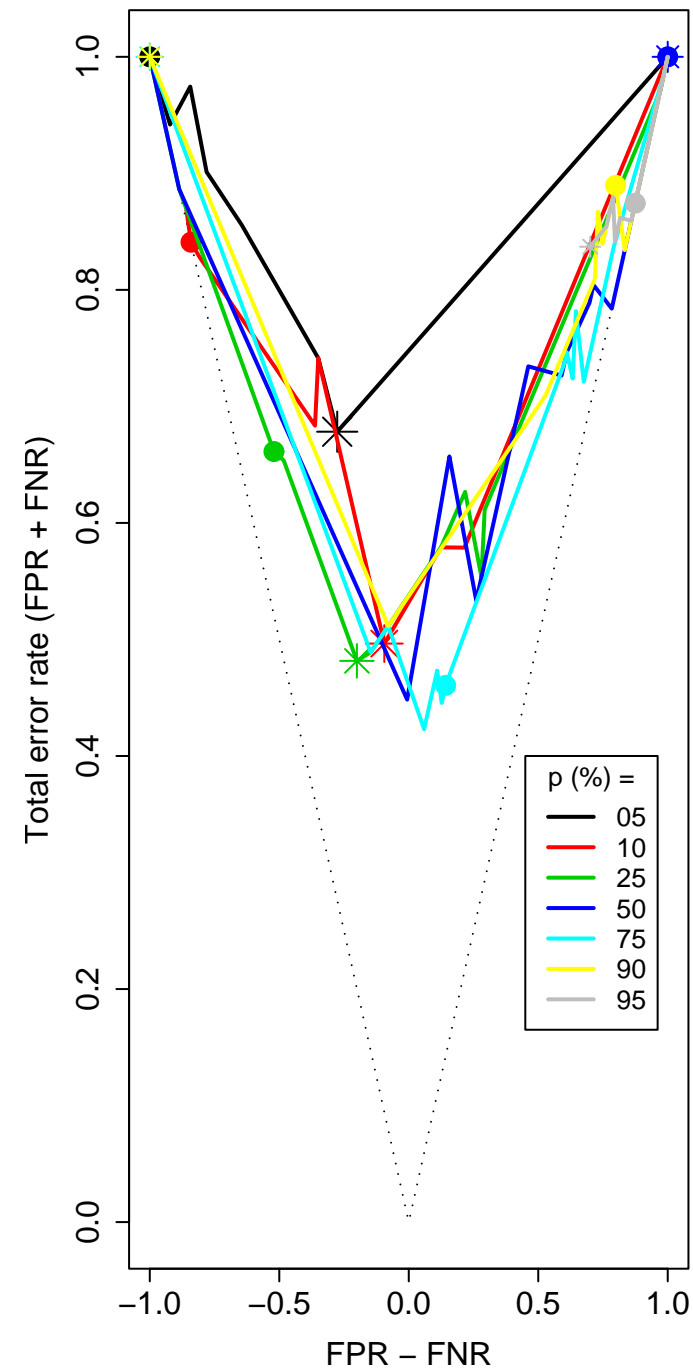

Supplement: Additional file 1 — Classifier performance vs class distribution for alleles A0203, A3101 and B1501. Comparison of the performance of classifiers built with training sets of same size but different proportions of positives for alleles A0203, A3101 and B1501 (compare to figure 2). Each point in a curve represents a classifier constructed with a different false negative training cost. The classifier constructed with the unit cost (λ1 = 1) in each curve is marked with a solid circle and that constructed with the balancing cost is marked with a star. The curve for the perfect classifier would lie on the dotted line. The y-axis shows the total error rate of a classifier, which is the same as the classifier cost (K) when the type 1 and type 2 misclassification costs are identical (κ1 = κ2 = 1). FNR: false negative rate. FPR: false positive rate. [file 1471-2105-9-385-S1.pdf]

**A0203**

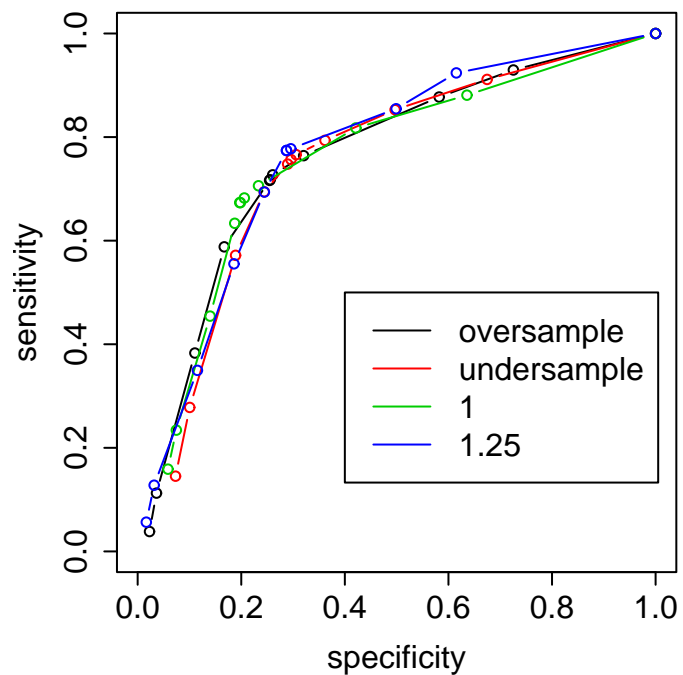

**A3101**

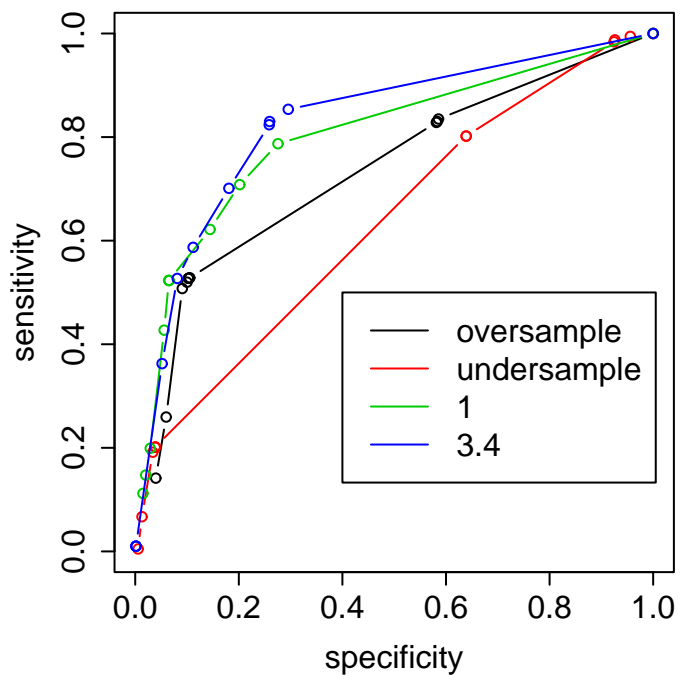

**B1501**

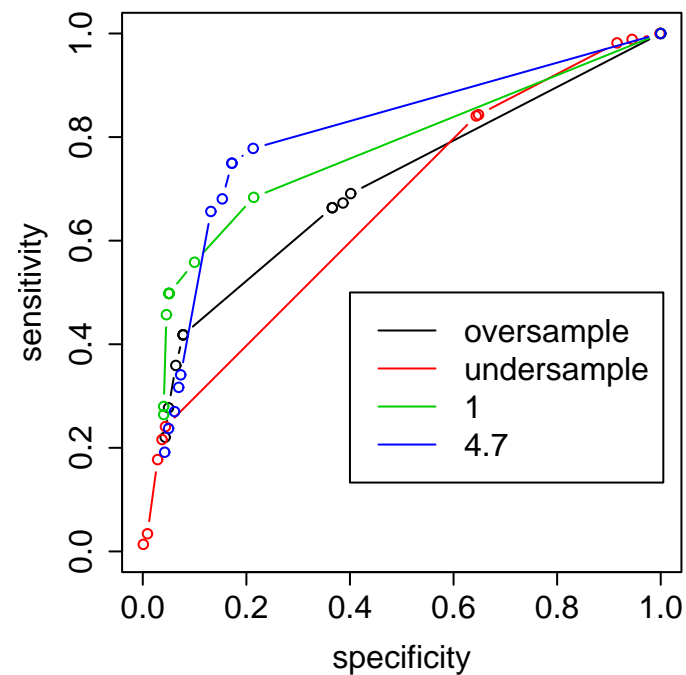

Supplement: Additional file 2 — Comparison of unit cost, balancing cost, undersampling and oversampling for alleles A0203, A3101 and B1501. ROC curves for alleles A1101 (left panel) and B0702 (right panel) comparing the results of trees constructed with the oversampled training set (black curve), the undersampled training set (red curve), and the full training set without training costs, that is, λ1 = λ2 = 1 (green curve) and with the balancing training cost, that is, λ1 = 1 and λ2 = (1 - p)/p (blue curve). Compare to figure 4. The ROC curves were constructed by varying the threshold used to label a node from 0 to 1 and evaluating its sensitivity and specificity at each threshold. [file 1471-2105-9-385-S2.pdf]
